# Prinicipal component analysis of myocardial strains to optimize cardiac resynchronization therapy patient selection

**DOI:** 10.1186/1532-429X-16-S1-O37

**Published:** 2014-01-16

**Authors:** Raghav Ramachandran, Frederick H Epstein, Kenneth C Bilchick

**Affiliations:** 1 Biomedical Engineering, University of Virginia, Charlottesville, Virginia, USA; 2 Department of Medicine, University of Virginia, Charlottesville, Virginia, USA

## Background

Cardiac resynchronization therapy (CRT) is effective for selected heart failure (HF) patients but improved methods for identification of mechanical dyssynchrony are needed. Cine displacement encoding with stimulated echoes (DENSE) MRI can be used to obtain high quality strain data for LV dyssynchrony quantification. An ideal dyssynchrony parameter should be time-independent and require minimal user interaction. We hypothesize that the time-independent, data-driven quantification of dyssynchrony using principal component analysis (PCA) will help improve patient selection for CRT.

## Methods

2D cine DENSE MRI was performed in multiple short-axis views to obtain LV circumferential strains (Ecc) for HF patients referred for CRT. In order to minimize confounding factors with an influence on CRT response, all patients in this analysis (N = 25) were required to have absence of scar as determined from late gadolinium-enhanced (LGE) MRI, as well as LV lead placement in a late-activated segment with time from QRS onset to the LV lead electrogram at the final lead position at least 50% of the QRS width. CRT response was defined as at least a 15% reduction in the LV end systolic volume 6 months after CRT implantation. Typical cine DENSE imaging parameters for HF patients include field of view = 340 - 400 mm^2^, matrix = 128 × 128, slice thickness = 8 mm, no. of spiral interleaves = 6, fat suppression, temporal resolution = 17 ms and displacement encoding frequency = 0.1 cycles/mm ^1^. The LV Ecc curves were decomposed spatially, using a 24-segment LV model, into principal component basis vectors. The PCA-based metric for measuring LV dyssynchrony, termed First Order Regional Conformity Estimate (FORCE), was calculated as |sum(PCL1)|/sum(|PCL1|) where PCL1 represents the loadings of the first principal component basis vector ^2^. The positive predictive value for FORCE was calculated using a threshold for LV dyssynchrony with no false negatives (sensitivity = 1). Receiver Operating Characteristic (ROC) analysis was performed to quantify the ability of FORCE to distinguish CRT responders from non-responders. Patient and LV functional/mechanical parameters were compared between CRT responders and nonresponders.

## Results

**Figure 1:**
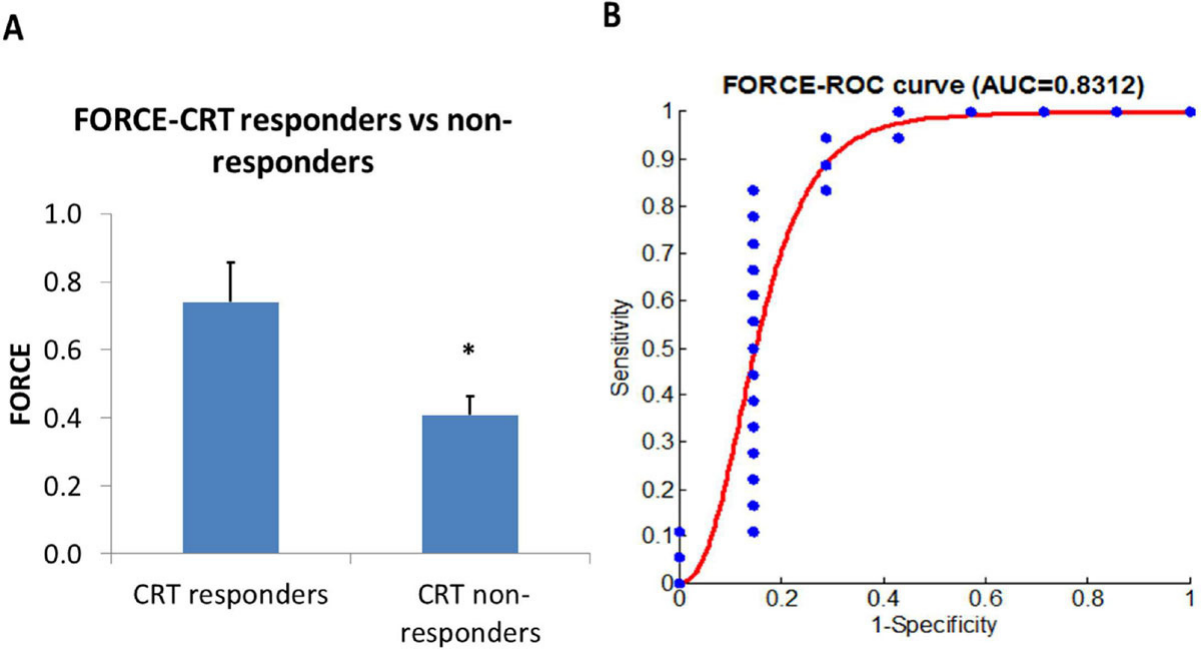
**A) Differences in FORCE between CRT responders and non-responders and B) ROC analysis of FORCE indicating its ability to identify CRT responders and non-responders**.

The overall CRT response rate was 72%. The comparison of FORCE between CRT responders and non-responders and the area under the ROC curve for FORCE (0.831) are shown in Figure 1. FORCE < 0.85 resulted in a positive predictive value of 0.818. Differences in LV function and patient parameters between CRT responders and non-responders are shown in Table 1.

**Table 1:** 

	CRT responders (18) Median (Interquartile range)	CRT non-responders (7) Median (Interquartile range)
Age (years)	61.4 (54.7 to 71)	69.9 (61.3 to 72.0)
Gender	66.7% female	14.3% female
% LV Ejection Fraction	24.5 (18.5 to 27.4)	26.7 (19.2 to 31.3)
% change in LV End-Systolic Volume index post-CRT	-37 (-51.9 to -22)	* 7.2 (-1.1 to 34.2)

* p-value < 0.05-Equal Variance Test

## Conclusions

Analysis of dyssynchrony using the novel data-driven and completely time-independent PCA-based metric FORCE is feasible and effective for prediction of CRT response in a select population with favorable lead placement and without LV scar. Further evaluation of this methodology is warranted in a broader heart failure population.

## Funding

This work was funded by the American Heart Association 12GRNT12050301, NHLBI K23 grant HL094761 and NIH T32HL007284.

## References

[B1] Jehle J Cardiovasc Magn Reson 2009

[B2] Ramachandran SCMR 2013

